# Loss to gain: pseudogenes in microorganisms, focusing on eubacteria, and their biological significance

**DOI:** 10.1007/s00253-023-12971-w

**Published:** 2024-05-08

**Authors:** Yi Yang, Pengzhi Wang, Samir El Qaidi, Philip R. Hardwidge, Jinlin Huang, Guoqiang Zhu

**Affiliations:** 1 College of Veterinary Medicine, Yangzhou University, 12 East Wenhui Road, Yangzhou, 225009 Jiangsu China; 2 Jiangsu Co-Innovation Center for Prevention and Control of Important Animal Infectious Diseases and Zoonoses, Yangzhou, 225009 China; 3 Joint Laboratory of International Cooperation On Prevention and Control Technology of Important Animal Diseases and Zoonoses of Jiangsu Higher Education Institutions, Yangzhou, 225009 China; 4 College of Veterinary Medicine, Kansas State University, Manhattan, KS 66506 USA; 5 Jiangsu Key Lab of Zoonosis, Yangzhou University, Yangzhou, 225009 Jiangsu China; 6 College of Bioscience and Biotechnology, Yangzhou University, 12 East Wenhui Road Yangzhou, Jiangsu, 225009 China

**Keywords:** Pseudogene, Microbiology, Pathogen, Pathogenicity, Evolution

## Abstract

**Abstract:**

Pseudogenes are defined as “non-functional” copies of corresponding parent genes. The cognition of pseudogenes continues to be refreshed through accumulating and updating research findings. Previous studies have predominantly focused on mammals, but pseudogenes have received relatively less attention in the field of microbiology. Given the increasing recognition on the importance of pseudogenes, in this review, we focus on several aspects of microorganism pseudogenes, including their classification and characteristics, their generation and fate, their identification, their abundance and distribution, their impact on virulence, their ability to recombine with functional genes, the extent to which some pseudogenes are transcribed and translated, and the relationship between pseudogenes and viruses. By summarizing and organizing the latest research progress, this review will provide a comprehensive perspective and improved understanding on pseudogenes in microorganisms.

**Key points:**

• *Concept, classification and characteristics, identification and databases, content, and distribution of microbial pseudogenes are presented.*

• *How pseudogenization contribute to pathogen virulence is highlighted.*

• *Pseudogenes with potential functions in microorganisms are discussed.*

## Introduction

Pseudogenes were first discovered as truncated and non-functional copies of the gene encoding 5S RNA in the genome of *Xenopus laevis* (Jacq et al. 1977). Many of pseudogenes have now been identified in all kingdoms of life, ranging from inferior organism to human and plants (Harrison et al. 2003; Torrents et al. 2003; Xie et al. 2019; Zhang et al. 2004). Pseudogenes are defined as defective nucleic acid sequences related to functional genes (or parent genes). In bacteria, pseudogenes, are defined here as “genes that have been silenced by one or more deleterious mutations” (Goodhead and Darby 2015). Relative to their functional homologs, pseudogenes are characterized by a variety of obvious defects in sequence, including insertion or deletion of nucleotides, frameshift, premature stop codons, and truncation. There are two ways to designate pseudogenes: One way is to mark the symbol “*ψ*” prior to the gene name, such as “*ψfepE*” (Hiyoshi et al. 2018), and the other way is to add the letter “*P*” after the gene name, such as “*CYP4Z2P*” (Zheng et al. 2016).

Initially, pseudogenes were regarded as “gene fossils” or “junk DNA,” but continued research has demonstrated biological function for many pseudogenes. Some pseudogenes produce a variety of functional RNA that play an important role in gene regulation and other physiological or pathological processes (Chen et al. 2020; Lai et al. 2019; Lou et al. 2020; Tam et al. 2008). These genes currently considered as pseudogenes, but these genes have potential functions. Some pseudogenes are also related to the occurrence, development, prognosis, and therapeutic targets of cancers (Chen et al. 2018; Kalyana-Sundaram et al. 2012; Kwon et al. 2021; Sisu 2021b; Sun et al. 2021).

Previous research on pseudogenes have mostly focused on mammals or plants, while studies or reviews on microbial pseudogenes are relatively rare. Initially, it was thought that microorganisms lacked or had very few pseudogenes. However, in recent years, the prevalence and importance of pseudogenes in microorganisms have gradually been recognized. In this review, we comprehensively summarize the history and recent progress in the field of microbial pseudogenes, including their concept, classification, characteristics, generation and fate, content, and distribution, as well as their impact on virulence. Additionally, consideration is also given to pseudogenes that may have potential functions. Further study of microbial pseudogenes will influence our understanding of pathogen virulence and molecular genetics.

## Classification and characteristics of pseudogenes

According to the sequence characteristics and mechanism of generation, pseudogenes can be divided into three major types; this includes duplicated pseudogenes, retro-pseudogenes, and circular RNA-derived pseudogenes.

### Duplicated (or unprocessed) pseudogene

**Fig. 1:**
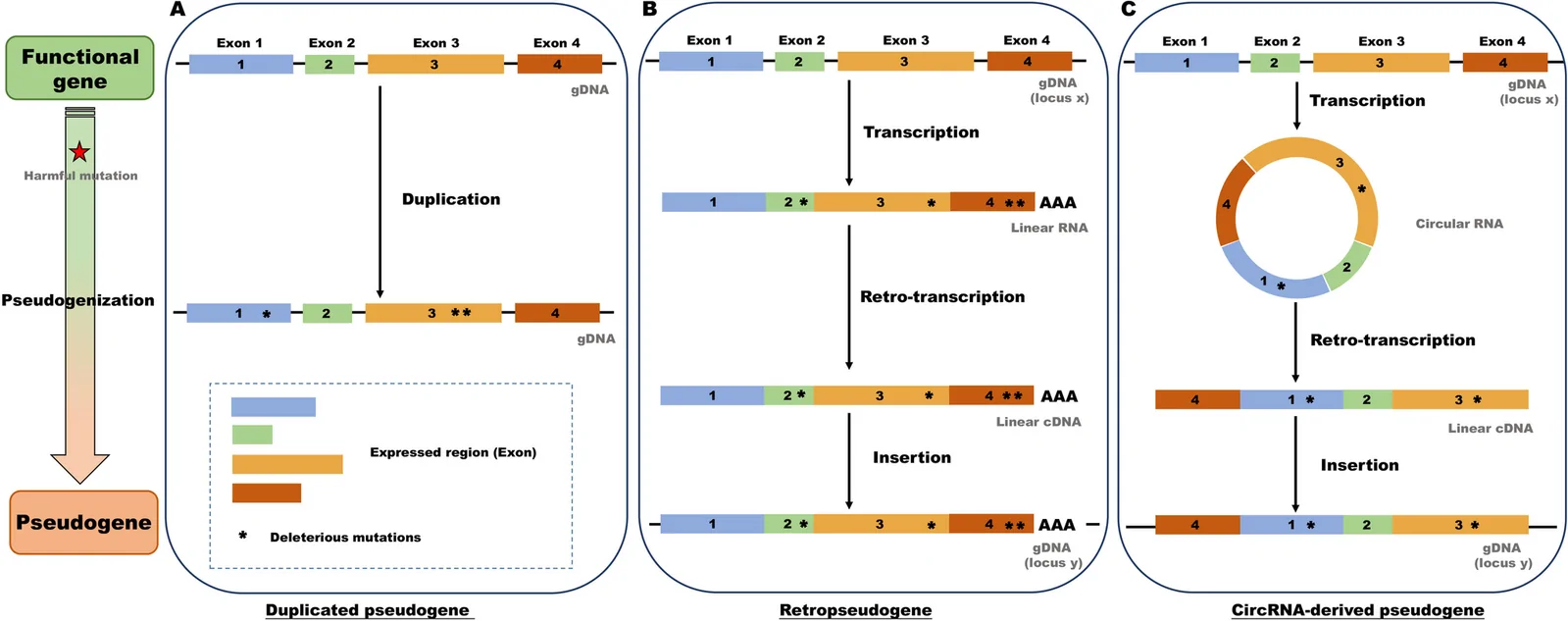
Cartoon illustration of different types of pseudogenes and pseudogenization. A schematic diagram of the **A** duplicated pseudogene, **B** retropseudogene pseudogene, and **C** the CircRNA-derived pseudogene

Pseudogene which arise from a gene duplication event and subsequent disabling mutation which is neither transcribed nor translated are called duplicated or unprocessed pseudogenes (Frankish and Harrow 2014). The deleterious mutations in nucleotide sequence include base deletion or insertion, premature stop codons and frameshift mutations, etc., which prevent the gene successful expression. Duplicated pseudogenes are very similar to parental genes in gene structure and retain much of the original sequence and structure, such as the promoter, intron, and exon (Fig. 1A) (Pei et al. 2012; Torrents et al. 2003).

### Retropseudogene (or processed) pseudogene

Pseudogene created by a retro-transposition event and subsequent disabling mutation which is neither transcribed nor translated are called retropseudogene or processed pseudogenes (Frankish and Harrow 2014). DNA is transcribed into mRNA and then reverse-transcribed into cDNA and re-integrated into the genome; during this process, the gene function is lost due to the inappropriate insertion site or the mutation of the sequence, resulting in the formation of pseudogenes. The features of retropseudogenes are as follows: (1) lack the region of introns; (2) lack the region of promoter (the randomness of the position of the retroposition inserted into the genome); (3) the flank contains untranslated region (UTR) structure, and the 3′ end contains a polyA tail (derived from mRNA, so it has characteristic structure of mRNA) (Fig. 1B) (Pei et al. 2012; Torrents et al. 2003).

### Circular RNA (CircRNA)–derived pseudogene

In addition to the above two pseudogenization mechanisms, researchers have recently discovered a new way of producing pseudogenes in mammals.

The pseudogene created by reverse transcription of circRNAs is called a circRNA-derived pseudogene (Dong et al. 2016). CircRNAs are a class of endogenous RNAs characterized by a covalently closed loop structure and without a 5′ cap and a 3′poly A tail (Verduci et al. 2021). Unlike linear RNAs, circRNAs are usually formed by reverse splicing events of exons or introns, that is, the downstream splicing site is connected to the upstream splicing site in reverse. Theoretically, a linear mRNA-derived pseudogene keeps the same sequential order (colinear) of exons as in its parent linear mRNA. In contrast, a circRNA-derived pseudogene can have an exon-exon junction in a reversed order (non-colinear). In addition, there is no polyA tail at the end of the sequence (Fig. 1C) (Dong et al. 2016; Li et al. 2018).

## Generation and fate of pseudogenes in microorganisms

### Generation of pseudogenes in microorganisms

DNA duplication and retro-transposition are the two main ways of generating pseudogenes in eukaryotes (Torrents et al. 2003). The formation of pseudogenes may differ between eukaryotes and prokaryotes. Phylogenomic analysis in prokaryotes indicated two mechanisms for pseudogene formation: (1) disruption of native genes and (2) failed horizontal gene transfer (HGT) events (Avni et al. 2018). The form of native gene disruption is diverse, including the point mutations, the presence of premature stop-codons, frame shift, etc. (Lerat and Ochman 2004). HGT is the process of an organism acquiring non-parental genetic information (Darmon and Leach 2014). HGT event between bacteria are common. HGT is one of the important mechanisms contributing to genetic diversity, microbial antibiotic resistance, survival, pathogenicity, and adaptation of bacteria (Darmon and Leach 2014; Soucy et al. 2015). It also plays an essential role in the evolution and speciation of bacteria (Darmon and Leach 2014). Liu et al. used a GC-content method to estimate horizontal transferred genes and reported that a large part of bacterial pseudogenes originated from failed HGT events (Liu et al. 2004).

### Fate of pseudogenes in microorganisms

Once established in a genome, pseudogenes evolve over time. In eukaryotes, pseudogenes often persist for long time during evolution. Moreover, some pseudogenes may be shared by distant relative lineages, such as rodents and primates (Zhang et al. 2004). However, reports reveal that, unlike eukaryotes, in bacteria, pseudogenes are usually deleted relatively rapidly from genomes, suggesting that their presence is deleterious to some extent (Kuo and Ochman 2010). During evolution, the continuous accumulation of mutations is one of the reasons for the exclusion of pseudogenes. Energy-consuming transcription or the detrimental product is also one of the reasons for the rapidly elimination of pseudogenes. Many other pseudogenes persist and evolve over time. Overall, the different evolutionary processes and selective pressures lead to retention or clearing of pseudogenes from the genome of microorganism over time.

## Identification of pseudogenes

Accurate identification and annotation of pseudogenes are the foundations and prerequisites of the research on pseudogene. Currently, the general process of pseudogene identification within the scope of genome is mainly as follows: (i) Firstly, a set of genome data is selected as the reference sequence; (ii) then, a group pseudogene is screened as candidate library based on their sequence homology with the parental gene, sequence defects, and the unique structural characteristics of pseudogenes, such as no exon structure and polyA tail structure; (iii) afterwards, incorrectly identified pseudogenes will be eliminated through different methods, such as manual inspection (Pei et al. 2012).

In fact, correctly distinguishing pseudogenes from parental genes is an extremely difficult process. It is worth noting that the misannotation of functional genes as pseudogenes can occur due to sequencing errors, which vary a lot depending on the sequencing method employed. By analyzing the mutation characteristics of known pseudogenes, a threshold can be deduced and utilized to distinguish between pseudogenes and functional genes.

### Pseudogene identification tools

Several bioinformatics tools are now available to predict pseudogenes. At present, commonly pseudogene identification methods include the following: PseudoPipe (Zhang et al. 2006), which was developed by the Gerstein team at Yale University, is suitable for identification of pseudogenes in mammals; PseudoFinder (Zheng et al. 2007) (annotation pseudogene based on the gene homology) and Retrofinder (Zheng et al. 2007) (focus on annotation of processed pseudogenes), which were developed by the University of California; and HAVANA (Human and Vertebrate Analysis and Annotation), which focus on manual annotation.

Moreover, sideRETRO, a pipeline dedicated to identifying processed pseudogenes, was developed in recent years (Miller et al. 2021). Abrahamsson et al. reported the PΨFinder (P-psy-finder), a tool that can identify processed pseudogenes from DNA sequencing data and predicts their location in the genome (Abrahamsson et al. 2022).

Besides, CIRCpseudo (Dong et al. 2016), which was conceived by Yang team from Shanghai Institute of Biological Sciences, Chinese Academy of Sciences, may be used to discover and identify potential pseudogene sequences derived from circRNAs in the genome.

Lerat et al. designed a suite of programs named “Ψ-Φ” (short for Ψ-gene Finder) to identify pseudogenes in bacteria genomes (Lerat and Ochman 2004). Recently, researchers published a new open-source software Pseudofinder that can be used for identification and analysis pseudogenes in in bacterial and archaeal genomes (Syberg-Olsen et al. 2022). Furthermore, new pan-genomic analysis tools such as PEPPAN can help to analyze and identify pseudogenes in the bacterial genome (Zhou et al. 2020b).

At present, obtaining the whole genome sequence of bacteria is convenient, but the annotation of pseudogene is delayed (Goodhead and Darby 2015). The challenges of many annotation tools of genome lead to pseudogene misannotation or nonrecognition (Lerat and Ochman 2004; Zhou et al. 2020a). Although some genes were identified and annotated as “pseudogenes” due to their pseudogene-like features, they may produce viable transcripts and proteins. In addition, new evidence is emerging regarding the potential expression and implication of pseudogenes in gene regulation. In the strict sense, such “pseudogenes” cannot be called pseudogenes by the definition. The determination of their functionality relies on the research conducted by the corresponding laboratory. In recent years, the development of novel biological information resources, such as Prokaryotic Genome Annotation Pipeline (PGAP) and Reference Sequence (RefSeq) project, has greatly improved the quality of prokaryotic genome annotation and the ability to distinguish pseudogenes (Li et al. 2021; Sayers et al. 2020; Tatusova et al. 2016).

### Pseudogene database

Some pseudogene databases have been built to collect, update, and publish pseudogene information, for example, psiDR (Pei et al. 2012), which was generated by GENCODE pseudogene resource project, is a consensus platform that integrates multiple pipelines. There are also other online available pseudogene databases, such as pseudoMap (Chan et al. 2013), PseudoFam (Lam et al. 2009), PseudoGene (pseudogene.org/), PseudoFuN (Johnson et al. 2019), and Dreambase (Zheng et al. 2018). Similar to eukaryotic genomes, the detection of pseudogenes within prokaryotic genomes relies on aligning them with parent genes and subsequently identifying sequence defects. Available databases for prokaryotic pseudogenes are mainly found in the “Prokaryote Pseudogene Information Site” (Liu et al. 2004).

In general, the factors affecting the quality of pseudogene annotations mainly include the following: (1) the quality of the reference genome. This is because pseudogenes may be incorrectly annotated if the reference genome is incorrectly annotated or contaminated; (2) quality of pseudogene identification pipeline and processes (Frankish and Harrow 2014). Different design principles and processes may lead to differences in the number of pseudogenes eventually identified and the accuracy of pseudogenes between different algorithms. Due to the different principles and processes of each pipeline, the results obtained by each pipeline may be various. For example, the number of pseudogenes eventually identified and the accuracy of annotation may be different. Furthermore, genome annotation may be challenging in some cases and might lead to confusing situations where real genes will be identified as pseudogenes and vice versa. However, there is so far no pipeline to detect pseudogenes that can take into account the requirement of both high-throughput and accuracy. The annotation accuracy can be improved by the following ways: selecting high-quality parent genome data as reference database and selecting multiple strategies combined with multiple algorithms for prediction. By summarizing and analyzing the limitations of existing algorithms, new prediction algorithms and processes can be improved and developed to help annotate, identify, and study more pseudogenes.

## Pseudogenes in microorganisms

Pseudogenes have been reported and well-studied mostly in mammalian genomes, such as human and mouse (Torrents et al. 2003; Zhang et al. 2004). Limited concern has been devoted into pseudogene studies in microorganisms. Previously, pseudogenes are generally believed to be rare in microbial genomes. In fact, pseudogenes have been found in a variety of microorganisms. For instance, in *Salmonella enterica* subsp. *enterica* serovar Typhi (*S*. Typhi), Parkhill et al. identified 204 pseudogenes from a gene complement of 4599 genes (Parkhill et al. 2001). In *Salmonella enterica* subsp. *enterica* serovar Typhimurium (*S*. Typhimurium), several pseudogenes corresponding to genes are known to contribute to virulence (Parkhill et al. 2001). A total of 95 and 101 pseudogene candidates in whole-genome of *Escherichia coli* (*E. coli*) strains K-12 and O157, respectively, were reported (Homma et al. 2002). Besides, pseudogenes sequences have been observed within the genome of other bacteria species, such as *Brucella* (Bialer et al. 2021), *Shigella* (Cervantes-Rivera et al. 2020), *Spirochete* (Liu et al. 2022a), *Staphylococcus* (Chieffi et al. 2020), and *Fungi* (Oh et al. 2021; Shimizu et al. 2021). In addition, abundant pseudogenes have also been found in the obligate parasite *Mycobacterium leprae* (*M. leprae*) (Cole et al. 2001; Silva et al. 2022), *Rickettsia* (Andersson et al. 1998; Liu et al. 2004), and *Anaplasma* (Lin et al. 2023). Meanwhile, pseudogenes have also been reported in *Archaea* (Badel et al. 2020).

### The content of pseudogenes

A comprehensive comparison and analysis data from 64 prokaryote genome sequences find a total of around 7000 pseudogene candidates, meanwhile indicating that pseudogenes are pervasive, ranging from 1 to 5% of the genome in most prokaryotes (Liu et al. 2004). These pseudogenes are associated with diverse protein families, such as ABC transporters, cytochrome P450 and PPE (proline-proline-glutamic acid) families (PF00067 and PF00823), and others that have a direct role in DNA transposition (Liu et al. 2004). The 64 genomes are divided into three categories by researchers: *Archaea*, pathogenic bacteria, and non-pathogenic bacteria, which include 3.6%, 3.9%, and 3.3% pseudogene in their genomes, respectively (Liu et al. 2004). However, another study indicated that pseudogenes appear to be more common in the genomes of pathogenic bacteria than in other non-pathogenic organisms (Lerat and Ochman 2005).

### Intracellular lifestyle

Some intracellular parasites contain more pseudogenes. In the genome of *M. leprae*, an obligate intracellular organism causing human leprosy, has a high proportion of pseudogenes (36.5%) (Liu et al. 2004). *M. leprae* has a relatively small genome (3.3 Mbp) compared to other mycobacterial species. The formation of pseudogenes eliminated many important metabolic pathways involved in siderophore production, oxidative and respiratory chains, lipid biosynthesis and metabolism, catabolic systems, and their regulatory circuits (Cole et al. 2001; Gomez-Valero et al. 2007; Liu et al. 2004; Sugawara-Mikami et al. 2022). *Neisseria meningitidis* also harbors a large number of pseudogenes (12.4%) (Liu et al. 2004; Schoen et al. 2009). In *Rickettsia*, pseudogenes account for a large fraction of the genome (9.7%) (Andersson et al. 1998; Liu et al. 2004).

### Host range

There are differences in the number and proportion of pseudogenes carried by pathogens with different host ranges. *Salmonella* is an important zoonotic pathogen with more than 2600 serotypes of different host ranges and clinical characteristics (Tanner and Kingsley 2018). Comparative genomic study of multiple *Salmonella* serotypes revealed that the number of pseudogenes is much higher in *Salmonella* with a narrow host range than that of *Salmonella* with a broad host range (Holt et al. 2009; McClelland et al. 2001). Johnson et al. reported that *S.* Typhi, a species of *Salmonella* restricted to humans, has a high proportion of pseudogenes (Johnson et al. 2018). One study reported that in *Salmonella enterica* subsp. *enterica* serovar Paratyphi A (*S.* Paratyphi A), a human-restricted pathogen that exists an average of 173 pseudogenes, while in *S.* Typhimurium, a general-host pathogen, approximately 30 pseudogenes were found (McClelland et al. 2004). Another study also showed a large number of genome degradation events through pseudogene formation in *S.* Paratyphi A (Jacob et al. 2023). A relatively high proportion of pseudogenes were also observed in the genome of *Salmonella enterica* subsp. *houtenae* (*S. houtenae*), an organism with host adaptation to reptiles (Hyeon et al. 2021). The observations obtained by comparing two *Salmonella* serotypes (*S*. Typhimurium and *S*. Enteritidis) with broad host ranges to two serotypes (*S*. Typhi and *S*. Pullorum) with limited host ranges are presented in Table 1. The analysis of the complete genome of *Staphylococcus aureus* (*S. aureus*) revealed the presence of 14 pseudogenes (range 2–30). In comparison, *S. aureus* subsp. *anaerobius*, an anaerobic subspecies restricted to sheep and goats, exhibited an average of 205 pseudogenes per genome (range 201–210) (Yebra et al. 2021).

**Table 1:** The proportion of pseudogenes in the genome of *Salmonella* with different host ranges

Host range	Serotype	Strain	Accession number ^1^	Genes (Total)^2^	Pseudogenes^2^	Proportion (%)	Average (%)^3^
Humans and many animals	*S*. Typhimurium	LT2	NZ_CP060507.1	4711	90	1.91	2.57 ± 0.44^**a**^
		ATCC 13311	NZ_CP009102.1	4690	120	2.56	
		NCTC 74	NZ_CP064709.1	4502	142	3.15	
		ATCC 14028	NZ_CP102669.1	4985	131	2.63	
		FDAARGOS_321	NZ_CP022070.1	4932	129	2.62	
	*S.* Enteritidis	P125109	NC_011294.1	4550	144	3.16	3.63 ± 0.35^**b**^
		SE006	NZ_CP099973.1	4586	159	3.47	
		CFSAN051827	NZ_CP075122.1	4679	169	3.61	
		ATCC BAA-708	NZ_CP025554.1	4622	188	4.07	
		18569	NZ_CP011394.1	4631	177	3.82	
Humans	*S*. Typhi	Ty2	NC_004631.1	4707	311	6.61	6.74 ± 0.30^**c**^
		WGS1146	NZ_CP040575.1	4826	341	7.07	
		S3	NZ_CP118537.1	4824	340	7.05	
		MDUST255	NZ_OU943337.1	4725	310	6.56	
		ISP2825	NZ_CP080960.1	4701	302	6.42	
Chicken	*S*. Pullorum	S06004	CP006575.1	4465	313	7.01	7.13 ± 0.29^**c**^
		ATCC 9120	NZ_CP012347.1	4654	336	7.22	
		QJ-2D-Sal	NZ_CP022963.1	4798	364	7.59	
		CDC1983-67	NC_022221.1	4476	311	6.95	
		R51	NZ_CP068386.1	4795	329	6.86	

^1^The sequence information of each strain is publicly available in the NCBI GenBank database and can be accessed using the accession numbers, which are listed in the fourth column of the table

^2^The number of total genes and pseudogenes of each strain is obtained directly from NCBI GenBank database

^3^Results are expressed as mean values standard deviation (SD). The data were evaluated using SPSS software (Version 27.0.1) through one-way analysis of variance (ANOVA) with the Tukey multiple comparison test for group comparisons. *P* values below 0.05 were considered significant. The presence of letters a, b, and c denotes statistically significant differences. Distinct letters indicate significant differences, while identical letters suggest non-significant differences

### Geographical environmental niche

In *Yersinia pestis* (*Y. pestis*), pseudogene accumulation may be a result of adaptive microevolution of the bacterium to different epidemic foci. For instance, pseudogene spectrum and genetic characteristics are different in *Y. pestis* strains from different epidemic foci (Tong et al. 2005). Based on this pseudogene distribution criteria, Tong et al. divided 260 *Y. pestis* isolates from different natural plague foci in China into eight genotypes (Tong et al. 2005). Therefore, pseudogene spectrum analysis can also be used as a typing technique for *Y. pestis*. Claesson et al. analyzed the genetic characterization of *Aggregatibacter actinomycetemcomitans* from periodontitis patients living in various geographical locations of Sweden (Claesson et al. 2022). They found that the genetic characteristics of isolates from different geographic areas were distinct. Moreover, based on the presence or absence of a point mutation in the pseudogene *hbpA2* of the bacterium, the collected isolates can be divided into two types: North African or West African. Current research evidence suggests that pseudogenes have potential applications in bacterial classification.

The accumulation of bacterial pseudogenes has previously been suggested to be associated with the adaptation of pathogens to specific host or ecological niches (Goodhead et al. 2020; Key et al. 2020; Liu et al. 2004). This partly explains why some genomes have a higher number of pseudogenes compared to others. When bacteria adapt from free living life style to an intracellular lifestyle, some genes, such as genes involved with metabolism or defense, become inactivated because their functions are no longer required in highly specialized niches (Cole et al. 2001). Some obligate intracellular bacteria such as *M. leprae* and *Rickettsia* have much smaller genomes than their free-living counterparts; some pathways tend to have been deleted (Dagan et al. 2006; Goodhead and Darby 2015). In addition, bacteria accumulate mutations and generate numbers of pseudogenes during the long-term process of adapting to different hosts or habitats (Dagan et al. 2006; Goodhead and Darby 2015; Wu et al. 2022). For example, Liao et al. found that the genomic characteristics vary among *Salmonella* populations from different hosts and geographic origins (Liao et al. 2020). Wang et al. identified that a unique glutathione (GSH) utilization pathway is pseudogenized in host-adapted *Francisella* (pathogenic *Francisella* species), whereas this pathway remains functional in non-pathogenic *Francisella* species, which are known to inhabit the environment (Wang et al. 2023). This possibility suggests that bacteria tend to eliminate and discard “unnecessary” genes, by forming pseudogenes, as they adapt to novel environmental niches, for example, when transitioning from a free-living lifestyle to an intracellular lifestyle, shifting from multiple hosts to unique host, or adapting to different epidemic foci (Bawn et al. 2020; Kuo and Ochman 2010). In general, pseudogenes are pervasively distributed in various microorganisms with different proportions. The formation and accumulation of pseudogenes in microorganisms are likely to be associated with the adaptive evolution of pathogens to specific niches, while other parameters may also play a role.

## Contribution of pseudogenization to pathogen virulence

**Fig. 2:**
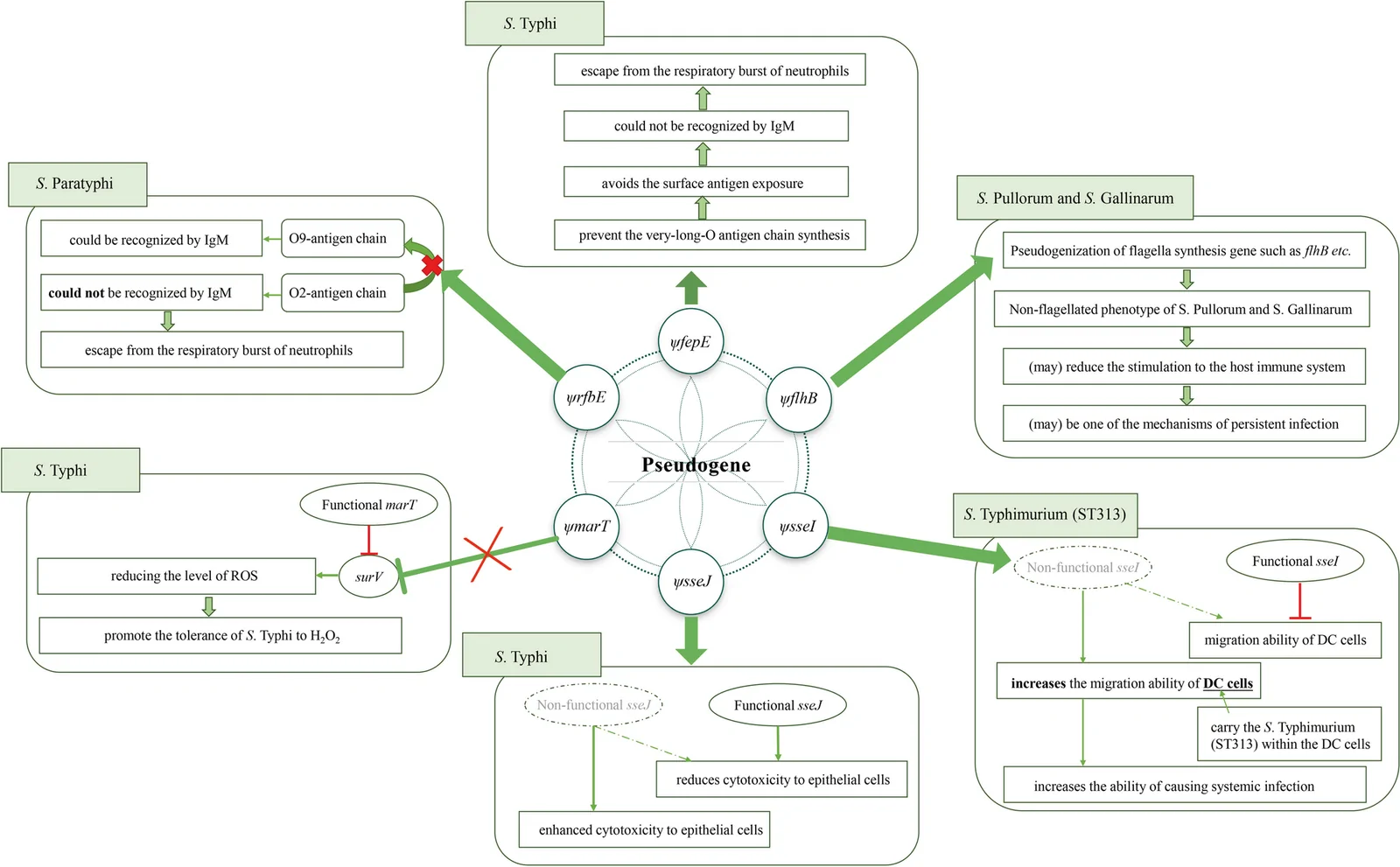
Implication of pseudogenes in oxidative stress response, bacterial infection, and evasion of host immune response in *Salmonella.* The examples include elucidating the mechanisms by which pseudogenization enhances virulence in *Salmonella*

Bacteria promote their virulence and environmental adaptation through gene acquisition and gene loss (Lawrence et al. 2001; Ochman and Moran 2001; Zhou et al. 2022). Acquisition of pathogenicity islands or virulence plasmids has been well studied. Pseudogenization is one of the most important mechanisms of gene loss, which is caused by the accumulation of nonsense mutations in protein-coding gene sequences (McCutcheon and Moran 2011). In the following section, we will discuss some mechanisms by which pseudogenization affects virulence in *Salmonella* (Fig. 2).

### Evasion of the phagocyte respiratory burst

**Fig. 3:**
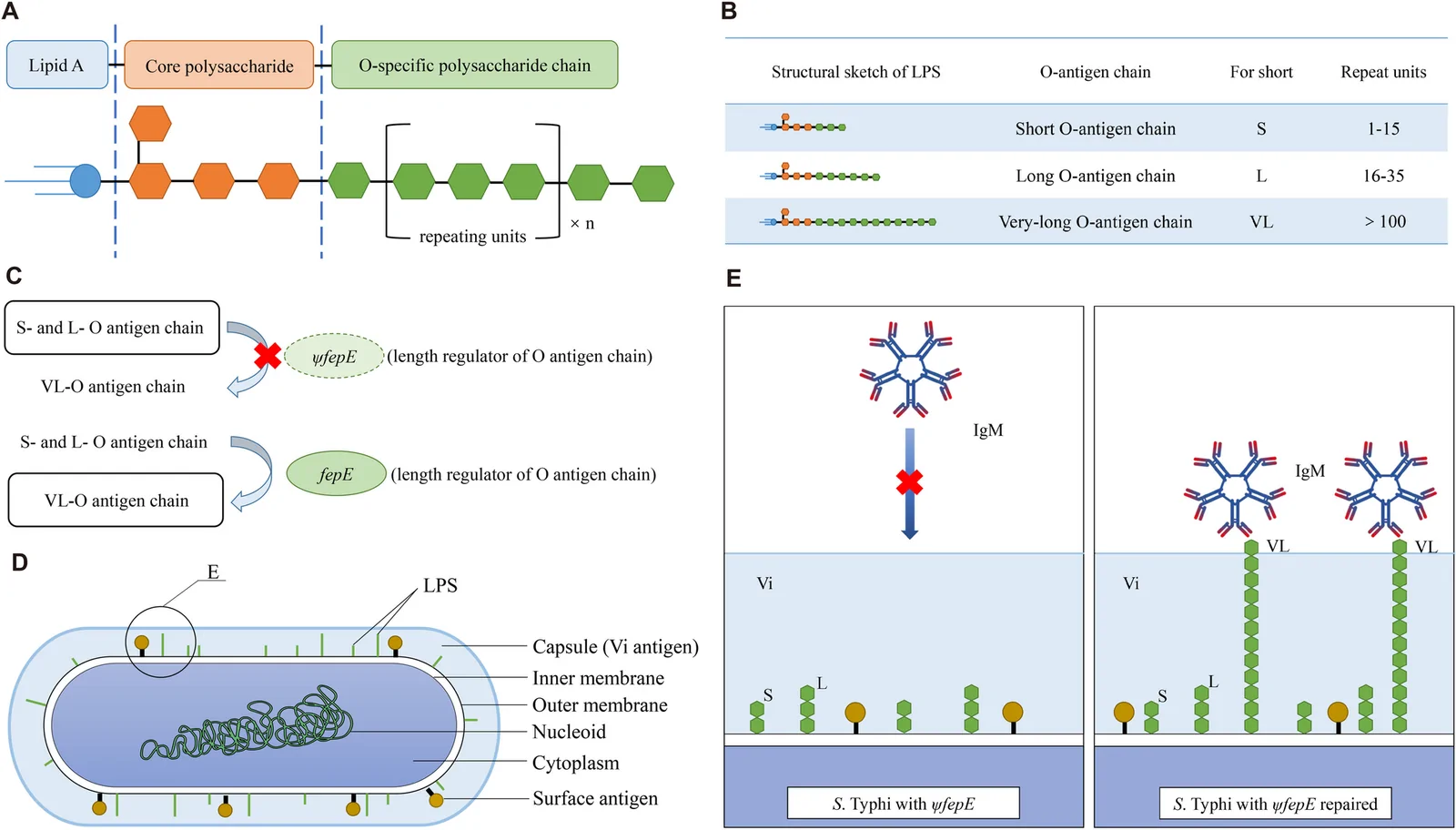
How pseudogenization of *fepE* optimizes capsule-mediated respiratory burst evasion by *S*. Typhi. **A** Structural schematic of LPS. **B** Three types of LPS according to the number of O-antigen repeat units. **C** Pseudogenization of *fepE* gene is responsible for the lack of LPS that with VL O-antigen chain in *S*. Typhi. **D** A diagram showing the brief composition and structure of *S*. Typhi. **E** A local magnification of the circular area in Fig. 3D. The capsule shields underlying surface structures from IgM recognition. Repair of *ψfepE* results in the production of LPS with VL O-antigen chain, which is exposed outside of the capsule, thereby resulting in IgM binding

Polymorphonuclear neutrophil leukocytes (PMNs) play an important role in phagocytosis and the killing of microbes. PMN respiratory burst, also known as oxygen burst, is an oxygen-dependent killing mechanism used to eliminate phagocytized pathogens. The activation of respiratory burst activates the NADPH oxidase, and a large number of reactive oxygen species (ROS) are produced (Luo et al. 2016). The capsule structure (Vi antigen) of *S*. Typhi prevents IgM-dependent complement activation and C5a-mediated neutrophil chemotaxis (Hiyoshi et al. 2018). *Salmonella* lipopolysaccharide (LPS) is composed of lipid A, the core polysaccharide, and an O-specific polysaccharide chain (O-antigen) (Fig. 3A) (Seif et al. 2019). The side chain of the O-antigen consists of several repetitive oligosaccharide units and can be divided into three types: (1) short (S) O-antigen chain, containing 1–15 repeating units; (2) long (L) O-antigen chain, containing 16–35 repeat units; (3) very-long (VL) O-antigen chain, containing more than 100 repeat units (Fig. 3B) (Murray et al. 2003). The length regulator FepE controls the length of the O-antigen chain. If the *fepE* gene is inactivated, the strain can only synthesize S or L O-antigen chains, but not the VL O-antigen chain (Fig. 3C) (Hiyoshi et al. 2018).

In *S*. Typhi, *fepE* gene is a pseudogene with an early termination codon in the coding gene sequence (Fig. 3C) (Crawford et al. 2013). After the pseudogene *ψfepE* from *S*. Typhi was repaired (the *fepE* gene from *S.* Typhimurium was transferred into *S.* Typhi), the mutant recovered the ability to synthesize LPS containing VL O-antigen chain. The presence of VL O-antigen chains stimulates a respiratory burst because parts of the O-antigen chain are exposed outside of the capsule (Fig. 3D) (Crawford et al. 2013).

**Fig. 4:**
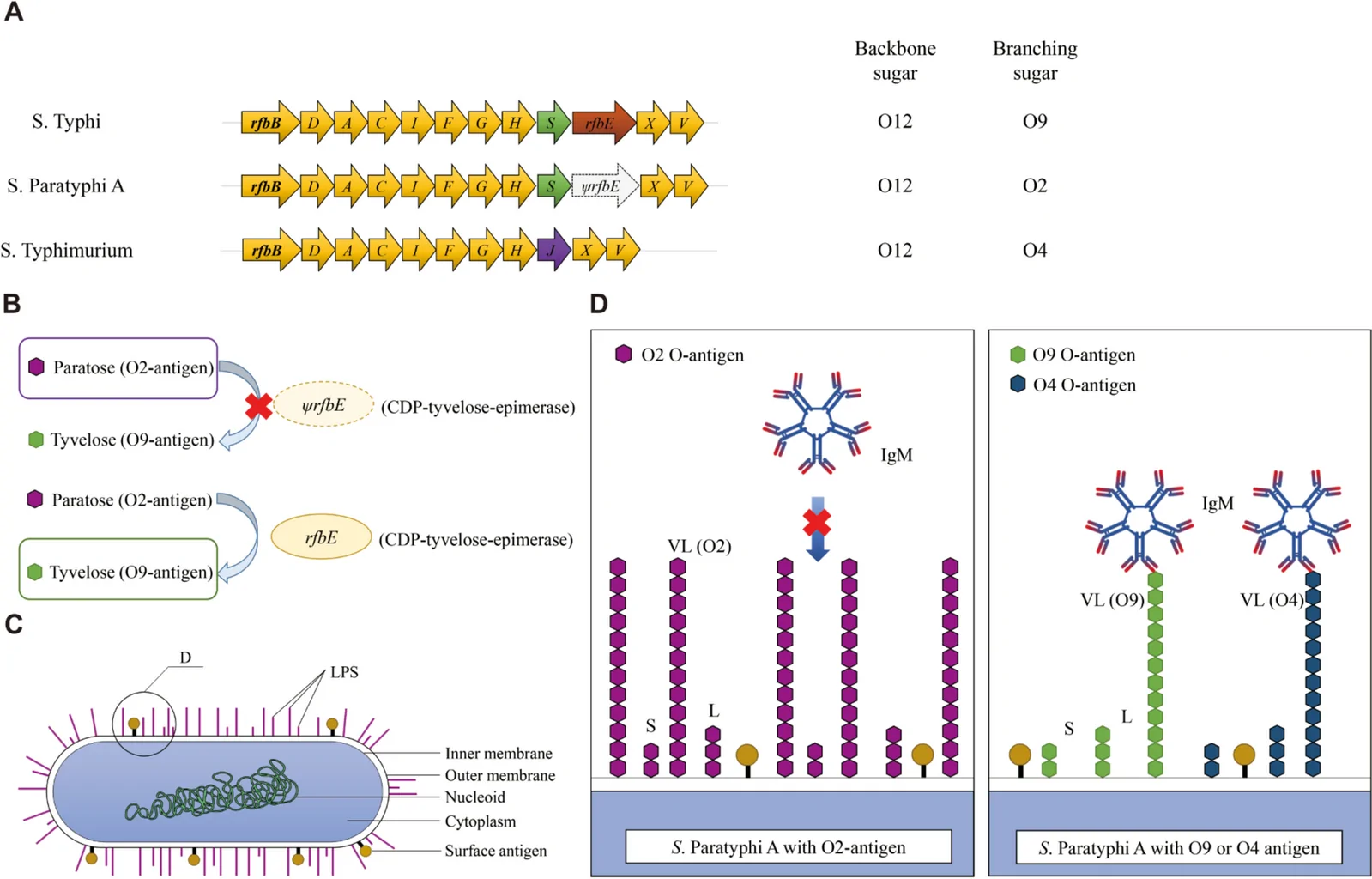
Pseudogenization of *rfbE* gene mediated respiratory burst evasion by *S*. Paratyphi A. **A** Gene clusters responsible for O-antigen biosynthesis in the indicated *Salmonella* serovars. **B** Pseudogenization of *rfbE* gene results in the production of O2 antigen in *S*. Paratyphi A. **C **A diagram showing the brief composition and structure of *S*. Paratyphi A. **D** A local magnification of the circular area in **C**. The VL LPS of *S*. Paratyphi A contains the O2 antigen which is not recognized by IgM. Mutants containing the O9 antigen (*ψrfbE* repaired) or the O4 antigen can be recognized by IgM

*S.* Paratyphi A can also escape from PMN respiratory burst, despite the lack of a capsule (McClelland et al. 2004). However, a rough mutant of *S.* Paratyphi A (var. Durazzo strain ATCC11511) lacks the VL O-antigen chain that triggers a strong respiratory burst (Hiyoshi et al. 2018). *S.* Paratyphi A contains the O2 and O12 antigens, with trisaccharide (mannose-rhamnose-galactose, O12) as the backbone and paratose (O2) as the branching sugar. *S.* Typhi contains the O9 and O12 antigens, with trisaccharide (O12) as the backbone and tyvelose (O9) as the branching sugar (Hiyoshi et al. 2018). *S.* Typhimurium contains the O4 and O12 antigens, with trisaccharide (O12) as the backbone and abequose (O4) as the branching sugar. The *rfbS* gene encodes a CDP-paratose synthase to catalyze the synthesis of paratose (O2). The *rfbE* gene encodes a CDP-tyvelose-epimerase to catalyze the conversion of paratose (O2) to tyvelose (O9). The *rfbJ* gene encodes a CDP-abequose synthase to catalyze the synthesis of abequose (O4) (Fig. 4A) (Verma and Reeves 1989; Woo et al. 2001). In *S.* Paratyphi A, the *rfbE* gene is annotated as a pseudogene because of mutations in the coding sequence. As a result, the conversion of paratose (O2) to tyvelose (O9) is not catalyzed, resulting in the production of O-antigen chain containing O2 rather than O9 (Fig. 4 B and C). The O2 antigen chain is the key *S.* Paratyphi A factor for inhibiting the host respiratory burst, as IgM binds to the O4 or O9 antigen but not to the O2 antigen. Introducing the *S.* Typhi *rfbE* gene into *S.* Paratyphi A results in O9 expression and triggers a respiratory burst. Replacing the *S.* Paratyphi A *rfbS* and *rfbE* genes with the *S.* Typhimurium *rfbJ* gene results in O4 expression and also triggers a respiratory burst (Fig. 4D) (Hiyoshi et al. 2018).

### Resistance to H_2_O_2_

The *marT* and *misL* genes are located in the *Salmonella* pathogenicity island (SPI-3). MarT is a transcriptional regulatory factor. MisL is a self-transporting adhesin that promotes the colonization of *S.* Typhimurium in the intestinal tract of mice. In *S.* Typhimurium, MarT acts as a transcriptional activator of MisL and directly promotes the transcription of *misL* (Dorsey et al. 2005). In *S.* Typhi, *marT* was annotated as pseudogene. The transfer of *marT* from *S.* Typhimurium into *S.* Typhi resulted in reduced tolerance to ROS (such as H_2_O_2_) (Ortega et al. 2016). SurV also promotes the tolerance of *S.* Typhimurium to H_2_O_2_ and is inhibited by MarT (Ortega et al. 2016; Retamal et al. 2010). However, the mechanism of how *surV* reduces ROS levels has not been fully clarified.

### Cytotoxicity toward epithelial cells

Some *Salmonella* type III secretion system (T3SS) effectors affect host cell cytotoxicity by altering *Salmonella*-containing vacuole (SCV) biosynthesis. SseJ is a T3SS-2 effector that functions as a cholesterol acyltransferase/lipase that alters SCV lipid content (Kolodziejek et al. 2019; Kolodziejek and Miller 2015; Walch et al. 2021). In *S.* Typhi, *sseJ* is annotated as a pseudogene *ψsseJ* (Trombert et al. 2010). The *S.* Typhimurium *sseJ* gene was introduced into *S.* Typhi, which were referred to as *S.* Typhi-*sseJ*^*S.*^ ^T^^yphimurium^. Polarized HT-29 epithelial cells monolayers were infected, and, compared with *S.* Typhi-WT, the ability of *S.* Typhi-*sseJ*^*S.*^ ^T^^yphimurium^ to destroy the HT-29 cell layer was reduced (Trombert et al. 2010). The destruction of the epithelial cell barrier function facilitates the spread of infections (Oscarsson et al. 2002). The functional *sseJ* appears to have a detrimental impact on the systemic infection of* S*. Typhi. Thus, the pseudogenization of *sseJ* enhances the cytotoxicity of *S*. Typhi towards epithelial cells and increases its virulence.

In a similar example, pseudogenization of *sopD2* enhances the pathogenicity of *S*. Typhi by increasing its ability to damage epithelial cells. SopD2 is also a T3SS-2 effector involved in SCV biosynthesis (Schroeder et al. 2010). In *S.* Typhi, *sopD2* is a pseudogene. When *S.* Typhimurium *sopD2* was expressed in *S.* Typhi, the toxicity of *S.* Typhi to epithelial cells was reduced and the ability of *S.* Typhi to penetrate epithelial cell also declined (Trombert et al. 2011).

### Systemic infection

*S.* Typhimurium has a wide range of hosts and generally causes limited gastroenteritis in humans and animals and scarcely causes systemic infections. Recently, typhoid *S.* Typhimurium strains causing systemic infections were isolated in sub-Saharan Africa (Feasey et al. 2012). The analysis of the isolates using multi-locus sequence typing (MLST) showed that their primary sequence type was sequence type 13 (ST313), whereas the typical *S.* Typhimurium strains associated with gastroenteritis mainly belonged to ST19 (Feasey et al. 2012; Kingsley et al. 2009). Compared with ST19, ST313 shows a certain degree of genomic degradation, including the formation of pseudogenes and gene deletions (Feasey et al. 2012; Kingsley et al. 2009). In a mouse infection model, ST313 *S*. Typhimurium has higher loads in tissues than that of ST19 type, and the same trend can also be observed in chickens (Parsons et al. 2013; Yang et al. 2015). *S.* Typhimurium D23580, a typical strain of ST313, can spread systemically, because of its ability to survive within the CD11b^+^ dendritic cells (DCs), and migrate with DCs from the intestinal tract through lymphatic system to lymphatic organs (Carden et al. 2017).

Some *S.* Typhimurium T3SS effector factors inhibit the migration of DCs. The *sseI* gene encodes a T3SS effector that inhibits the migration of DCs in isolates associated with gastroenteritis. The *sseI* gene is a pseudogene in the genome of D23580 (Carden et al. 2017). The expression of functional *sseI* in *S*. Typhimurium D23580 decreased the migration ability of DCs, reduced *Salmonella*-carrying DC cells in mesenteric lymph nodes and decreased the bacterial loads in mesenteric lymph nodes. When the *sseI* gene was deleted in *S*. Typhimurium ST19, the number of bacteria that migrated to mesenteric lymph nodes increased (Carden et al. 2017). Therefore, the pseudogenization of the *sseI* gene enhances the ability of ST313 *S.* Typhimurium to cause systemic infection.

Additionally, the *sopA* gene encodes SopA, a T3SS-1 effector that functions as a HECT-like E3 ubiquitin ligase and is associated with *Salmonella*-induced enterocolitis. The recent study demonstrated that the pseudogenization of the *sopA* gene favored the survival of human macrophages and facilitated systemic infection of *S*. Typhi (Ma et al. 2021).

### Flagella formation

Flagella are important virulence factors of bacteria, which not only provide bacteria with mobility but also play crucial roles in adhesion, invasion, and biofilm formation during the process of bacterial pathogenicity (Duan et al. 2013, 2012; Zhou et al. 2013, 2015). It is thought that all *Salmonella* have flagella, except for *Salmonella enterica* subsp. *enterica* serovar Gallinarum (*S.* Gallinarum) and *Salmonella enterica* subsp. *enterica* serovar Pullorum (*S.* Pullorum). Although *S.* Gallinarum and *S.* Pullorum do not have flagellar structures on the bacterial surface, they possess a full set of flagellar-related coding genes. Some of those flagella-related coding genes are defined as pseudogenes in *S.* Gallinarum and *S.* Pullorum, while in other flagellated *Salmonella* genomes, the corresponding genes are functional and do not correspond to pseudogenes (Yang et al. 2020).

Among the many serotypes of *Salmonella* with flagella, we selected *S*. Enteritidis strain P125109 (GenBank Accession Number: AM933172.1), which has the nearest genetic relationships with *S*. Pullorum and *S.* Gallinarum, as the reference strain. Eight genomic sequences of *S*. Gallinarum and *S*. Pullorum, including *S*. Gallinarum str. 287/91 (AM933173.1), *S*. Gallinarum str. 9184 (CP019035.1), *S*. Gallinarum str. 9 (CM001153.1), *S.* Pullorum str. S06004 (CP006575.1), *S*. Pullorum str. ATCC9120 (CP012347.1), *S.* Pullorum S44987_1 (LK931482.1), *S*. Pullorum str. CDC1983-67 (CP003786.1), and *S*. Pullorum str. RKS5078 (CP003047.1), were obtained from the GenBank database. We compared the flagella-related gene sequences of *S.* Pullorum and *S.* Gallinarum with reference strain and then summarize nine pseudogenes associated with flagella synthesis in the genome of *S.* Pullorum and *S.* Gallinarum: *ψflgK*, *ψflhB*, *ψflhA*, *ψflgI*, *ψcheM*, *ψfliN*, *ψfliL*, *ψmotB*, and *ψycgR* (Table 2). If the gene is annotated as a pseudogene in the GenBank database, it is marked with “□”; and if it is a functional gene, it is marked with a “■.”The functions of each gene are listed under the gene name in Table 2, according to their annotation in the genomic database. Each *S.* Pullorum or *S*. Gallinarum strain contains at least two flagellar-related pseudogenes and up to six flagellar-related pseudogenes. Among them, *ψflgK* and *ψflhB* were pseudogenes in all *S.* Pullorum and *S.* Gallinarum strains.

**Table 2:** Distribution of flagella-associated pseudogenes in various *S*. Gallinarum and *S*. Pullorum strains

Strain	Flagella-associated genes/pseudogenes									Accession No.
	*flgK*	*flhB*	*flhA*	*flgI*	*cheM*	*motB*	*fliN*	*fliL*	*ycgR*	
	Hook associated protein	Export apparatus protein B	Export apparatus protein A	P-ring protein	Chemotaxis protein	Motor protein B	Motor switch protein	Basal Body protein	c-di-GMP binding protein	
*S.* Enteritidis str. P125109^a^	■	■	■	■	■	■	■	■	■	NC_011294
*S.* Gallinarum str. 287/91	□	□	□	□	□	■	■	■	■	AM933173.1
*S.* Gallinarum str. 9184	□	□	□	□	□	■	■	■	■	CP019035.1
*S.* Gallinarum str. 9	□	□	□	□	□	■	■	■	■	CM001153.1
*S.* Pullorum str. S06004	□	□	□	■	■	□	□	□	□	CP006575.1
*S.* Pullorum str. ATCC 9120	□	□	■	■	■	□	■	■	■	NZ_CP012347.1
*S.* Pullorum S44987_1	□	□	■	■	■	■	■	■	■	LK931482.1
*S.* Pullorum str. CDC1983-67	□	□	■	■	■	■	■	■	■	NC_022221.1
*S.* Pullorum str. RKS5078	□	□	■	■	■	■	■	■	■	CP003047.1

The functions of each gene are listed under the gene name in the table, according to their annotation in the genomic database. “□” represents the gene was annotated as a pseudogene in the genomic database; “■” represents the gene was functional gene

^a.^*S.* Enteritidis P125109 serves as a reference strain

FlgK, also known as HAP1 (hook-associated protein 1), is responsible for the effective connection between the flagellum hook and the flagellum in the process of flagella assembly (Makishima et al. 2001; Minamino et al. 2021). FlhB is located at the bottom of flagellar base and is responsible for controlling substrate export (Ferris et al. 2005; Halte and Erhardt 2021; Kuhlen et al. 2020). It remains unclear how the pseudogenization of the flagella genes benefits *S*. Pullorum and *S*. Gallinarum. It is known that the major structural protein of flagella, flagellin (FliC), is an agonist of Toll-like receptor 5 (TLR-5) (Duan et al. 2013). Given this, the non-flagella phenotype of *S*. Pullorum and *S*. Gallinarum probably confers an advantage in evading recognition by TLR-5 and attenuating the host immune response directed towards them.

In addition to *Salmonella*, there are also instances in which the pseudogenization of certain genes benefits the pathogenicity in other pathogens. The *yadE* gene is a pseudogene in *Y. pestis* and is functional in *Yersinia pseudotuberculosis* (*Y. pseudotuberculosis*). The *yadE* gene encodes YadE, a trimeric autotransporter that contributes to the stability of biofilm in *Y. pseudotuberculosis*. The expression of functional *yadE* in *Y. pestis* results in a reduced biofilm stability and altered production of Hms-ECM, which is important extracellular matrix for *Y. pestis* biofilm formation. The formation of biofilm is contributing to the survival, host interaction, and transmission of *Yersinia*. Therefore, the pseudogenization of *yadE* gene likely plays a role in the virulence of *Y. pestis* (Calder et al. 2020).

## Functional pseudogenes

For ages, pseudogenes have been considered as dysfunctional copies of functional genes; however, emerging evidence suggests that many of them may be biologically active (Cheetham et al. 2020). Pseudogenes may play physiological roles, such as gene expression, gene regulation, generation of genetic (antibody, antigenic, and other) diversity, as proposed and reported in mammals, such as humans and mice (Balakirev and Ayala 2003; Sisu 2021a; Xu and Zhang 2016). In the field of microbiology, the evidence that pseudogenes are not “completely non-functional” is mainly related to the fact that pseudogenes are involved in recombination and improve genetic diversity. Additionally, in some cases, they can be transcribed and translated.

### Contribution to gene genetic diversity

An important function of pseudogenes is to serve as a repository, providing material for improving gene sequence diversity. Chicken antibody (immunoglobulins, Igs) diversity is a classic example. Antibodies consist of two light chains (L) and two heavy chains (H), both of which contain a constant region (C) and a variable region (V), which is used to recognize foreign molecules (Ratcliffe 2006). The pseudogenes ψVL and ψVH are present upstream of the site of the coding nucleic acid sequence encoding the heavy and light chains of the antibody. Although the pseudogene itself cannot be expressed, it can be recombined with the variable region within the functional gene through insertion or replacement and expressed together with the functional gene, thereby increasing the diversity of the variable region coding gene (Vihinen 2014).

Antigenic variation is a strategy for the persistence of multiple microbial pathogens within hosts. Some pathogens use pseudogenes to produce antigenic variation in surface molecules, which is another example of using pseudogenes to significantly increase genetic diversity. For instance, pseudogene-mediated antigen variation is an important strategy for pathogenic microorganisms to evade host immune responses. *Trypanosoma brucei* (*T. brucei*) is a type of zoonotic parasite that causes sleeping sickness in human and Nagana disease in animals. *T. brucei* escapes the host’s immune system by periodically altering the variant surface glycoprotein (VSG) which covers the surface of the worm body. This variation relies on the large number (~ 2000) copies of *vsg* gene and the pseudogene form of *vsg* gene transcript (*ψvsg*) in the genome (Dakovic et al. 2023; Davies et al. 2021). As a result, the excessive level of recombination events between “*vsg*–*vsg*” and “*vsg*–*ψvsg*” continuously generates additional chimeric *vsg* genes and further enhances the diversity and richness of the *vsg* gene (Chandra et al. 2023; Faria et al. 2022).

Similar mechanisms have also been found in other pathogens. For example, both the major surface protein 2 (Msp2) of *Anaplasma* (Graca et al. 2019; Liu et al. 2019; Rejmanek et al. 2012) and the variable major protein (VMP) of *Borrelia* spp. (Gilmore et al. 2023; Restrepo et al. 1994; Schwartz et al. 2021) use the “functional gene-pseudogene” recombination mechanism to generate new variant antigens, thereby evading the immune recognition of hosts. In *Pneumocystis jirovecii*, which is an obligate pulmonary pathogen in human, pseudogenes are suggested to contribute to the generation of various mosaic *msg* genes, encoding the major surface glycoprotein, via integration into functional *msg* genes (Schmid-Siegert et al. 2017). Thus, it is obvious that “gene-pseudogene rearrangement” is an original genetic evolutionary mechanism, which contributes to the generation of genetic variability and diversity. Simultaneously, the presence of pseudogenes could serve as a reservoir of sequences for antigenic variation. Furthermore, in this case, the pseudogenes would not be eliminated or lost from the genome.

### Some pseudogenes are transcribed or translated

Due to the development of next-generation sequencing (NGS) technologies, the cost of large-scale sequencing has largely reduced. It is easier to obtain high-throughput genome-wide sequence data (Ejigu and Jung 2020; Rehder et al. 2021). Pseudogenes have been discovered in large numbers from the genomics data of various organisms, with the aid of NGS technologies. Concurrently, data from transcriptomics and proteomics techniques are improving our understanding of whether pseudogenes are expressed at the RNA/protein level or not. Transcription and translation of pseudogenes have been widely reported and confirmed in mammals (Giuliani et al. 2022; Qian et al. 2022; Sisu et al. 2020), while the transcription or expression of pseudogenes has also been gradually observed and reported in microorganisms. RNA-seq technique was used to analyze pseudogenes in *S*. Typhi; results reveal that many pseudogenes are transcribed, despite at greatly reduced levels (Perkins et al. 2009). Feng et al. utilized RNA-seq and mass spectrometry technologies to describe the transcriptional and translational landscape in *Salmonella* species. They revealed that 101 out of 161 pseudogenes could be successfully translated in *S.* Paratyphi A and *S*. Typhi (Feng et al. 2022). Transcribed pseudogenes have also been observed in *Shigella flexneri* (Cervantes-Rivera et al. 2020; Chanin et al. 2019). Whole-genome analysis of *M. leprae* RNA expression demonstrated that pseudogenes and non-coding regions are not silent but strongly expressed (Akama et al. 2009). Gao et al. analyzed the proteome of *Saccharomyces cerevisiae* using mass spectrometry (MS) and provided evidence that pseudogenes can be translated (Gao et al. 2021). Goodhead et al. applied multiple “omic” strategies, combining genomics, transcriptomics, and proteomics techniques to analyze the transcriptional and translational landscape of pseudogenes in *Sodalis*, a Gram-negative, facultative endosymbiont bacterium. They have revealed that *Sodalis* pseudogenes are often transcribed, but at a significantly lower level than intact CDSs (Goodhead et al. 2020). Wen et al. reported the observation of a set of small interference RNAs (siRNAs) derived from pseudogenes of African *T. brucei* using high-throughput analysis. Authors then confirmed that the siRNA derived from pseudogenes in the *T. brucei* can regulate protein-coding gene expression by means of the RNA interference (Wen et al. 2011).

Some pseudogenes have a role in aspects of bacterial diseases pathogenicity. Adhesins are located on the bacterial surface and help bacteria to adhere to the surface of host cells (Duan et al. 2022; Patel et al. 2017). In *Salmonella*, the *shdA* gene encodes a novel non-pilus autotransporter adhesin in which the passenger domain binds to one or more extracellular matrix proteins (e.g., fibronectin and collagen) (Kingsley et al. 2004, 2002; Paxman et al. 2019). In *S.* Typhi, the deletion of a *shdA* gene fragment causes a termination codon to be prematurely generated, therefore, it was annotated as a pseudogene (*ψshdA*) (Urrutia et al. 2014). However, subsequent experiments have shown that the translation product of *ψshdA* can function as an active adhesin, based on the analysis of a *ψshdA* deletion as well as the detection of a translated protein (Urrutia et al. 2014). Furthermore, gene *yqiG* is annotated as a pseudogene (*ψyqiG*) in *E. coli* BW25113 genome due to the insertion of an insertion sequence (IS) element inside the gene sequence. However, subsequent experiments have shown that the product of pseudogene *ψyqiG* (YqiG protein) is functional and was identified as an essential protein in glycolysis pathway and hydrogen metabolism in *E. coli* BW25113 (Zakaria et al. 2018). Zhang et al. reveal that the pseudogene BMEA_B0173 plays a role in the virulence of the *Brucella melitensis* (Zhang et al. 2022). In a mouse infection model, the BMEA_B0173 deletion mutant exhibited increased colonization in the spleen compared to the wild-type strain.

## Pseudogenes and viruses

### Are there pseudogenes in the viruses?

Do the viruses contain pseudogenes or not? Some studies have reported the existence of defective mutated genes in a variety of viral genomes (Ceccaldi et al. 1998; Zhang et al. 1992); hence, these mutated genes, which, by definition, should be called pseudogenes. Aaskov et al. reported that there is a stop-codon mutation in the surface envelope (E) protein gene of dengue virus type 1 (DENV-1) in humans and mosquitoes from Myanmar (Aaskov et al. 2006). Hughes et al. found that mutations were present in the genome of the equine influenza virus (EIV) and cause stop codons (Hughes et al. 2012). Some studies have shown that there are large numbers of nonfunctional genes (pseudogenes) in human cytomegalovirus (HCMV) strains (Sijmons et al. 2014, 2015; Suarez et al. 2019). One study shows that approximately 75% of HCMV virus strains carry pseudogenes (Sijmons et al. 2015; Suarez et al. 2019). The researchers also point out that the evidence for pseudogenes was largely derived from strains isolated in cell culture. These mutations are substitutions that introduce premature stop codons or deletions or insertions that cause frame shifting (Suarez et al. 2019).

### Pseudogenes and virus characterization

Do the pseudogenes contribute to alterations in virus characteristics? Up to now, the research about this aspect previously is not much. It has been reported that the mutations in the DENV-1 virus causing stop codons were likely to be long-term transmitted, and these mutations could provide the shift of viral fitness and then influence transmission dynamics (Aaskov et al. 2006). Here we attempt to explore the relationship between the gene defect mutations (pseudogenes) and the virulence of the virus, taking the severe acute respiratory syndrome coronavirus 2 (SARS-CoV-2) as an example.

The outbreak of COVID-19, which was caused by the severe acute respiratory syndrome coronavirus 2 (SARS-CoV-2), has a global impact. SARS-CoV-2 is an enveloped, positive-sense, single-stranded RNA virus and belongs to lineage B of the β-coronavirus genus. The virus genome encodes four structural proteins including spike (S), envelope (E), membrane (M), and nucleocapsid (N) proteins, as well as other non-structural or accessory proteins (Gordon et al. 2020). The genetic changes of the virus have attracted widespread and persistent attention around the world (Mannar et al. 2022). During the genomic surveillance of SARS-CoV-2 in the first year of the COVID-19 pandemic, a mutation that aspartic acid (D) was substituted by glycine (G) at position 614 (D614G) in the S protein was reported (Korber et al. 2020; Yurkovetskiy et al. 2020). The SARS-CoV-2 variant carrying the D614G mutation has then become the most prevalent form in the global pandemic. D614G shifts the conformation of the S protein and alters the affinity toward to the angiotensin-converting enzyme 2 (ACE2), which is the receptor of SARS-CoV-2 (Yurkovetskiy et al. 2020; Zhang et al. 2021a). D614G increased the infectivity of human lung cells or bat or pangolin ACE2 cells (Yurkovetskiy et al. 2020). The D614G variant was proved to have the ability of efficient replication in vitro and transmission in vivo (Hou et al. 2020; Shi and Xie 2021).

In addition, several defective mutations in the SARS-CoV-2 genome have been reported to date. Su et al. reported that there is a 382-nucleotide (nt) deletion (Δ382) in open reading frame ORF7b and ORF8 of SARS-CoV-2 (Su et al. 2020). This mutation resulted in truncation of ORF7 and non-transcription of ORF8 (Su et al. 2020). The Orf8 encodes an accessory protein and is one of the proteins that with least sequence similarity between SARS-CoV-2 and SARS-CoV (Gordon et al. 2020; Zhang et al. 2021b). ORF8 protein was be proved to mediate immune evasion through down-regulating major histocompability complex class Ι (MHC-Ι) (Zhang et al. 2021b). Recent studies revealed that ORF8 could mediate endoplasmic reticulum (ER) reshaping of host cell through forming mixed disulfide complexes with ER proteins (Liu et al. 2022b). Clinical symptom of patients infected with ∆382 variant viruses was milder, compared to patients infected with the wild viruses (Young et al. 2020). In addition, lower concentrations of proinflammatory cytokines were detected in plasma of patients infected with ∆382 variant viruses, compared to that of patients infected with the wild viruses (Young et al. 2020). In summary, it seems that ORF8 could be a candidate target for the development of treatments and vaccines for SARS-CoV-2.

Researchers reported that there is a premature stop codon in ORF3b gene at position 14 (E14*) of SARS-CoV-2 (Gordon et al. 2020). This mutation resulted in truncated product of ORF3b (Konno et al. 2020). This mutation resulted in truncated product of ORF3b (Konno et al. 2020). The Orf3 encodes one of the accessory proteins of SARS-CoV-2. Qu et al. reported that ORF3a mediated incomplete autophagy, which facilitates SARS-CoV-2 replication (Qu et al. 2021). The antibodies of protein ORF8 and ORF3b are accurate serological markers of early and late SARS-CoV-2 infections (Hachim et al. 2020). ORF3b of SARS-CoV-2 is a potent IFN-I antagonist, which inhibited the activation of human IFN-I (Konno et al. 2020). Besides, researchers reported that there are two premature stop codons at positions 41 (Q41*) and 44 (Q44*) in ORF9c of SARS-CoV-2 (Gordon et al. 2020). The biological significance of mutant in SARSCoV-2 ORF9c remains to be elucidated. Further research focused on defective gene variants (pseudogenes) of the virus may improve the understanding of the mechanisms of virus infection and could provide new insights in the development of targets for treatments and vaccines.

### Virus-derived pseudogenes in mammals

The investigation and analysis of mammalian genomes suggest that some pseudogenes in mammals may be derived from viruses. For instance, a study analyzed the *Mops condylurus* genomic DNA samples and revealed that there is an Ebola virus nucleoprotein (NP)-derived pseudogene inserted in its genome (Hermida Lorenzo et al. 2021). Furthermore, filovirus-derived pseudogenes have also been reported in the genome of a wide variety of organisms including bats, marsupials, and rodents (Taylor et al. 2011). This is thought to originate from recombination events between viral genes and host genomic transposons during viral infection (Naville et al. 2016), while the significance and importance of the virus-derived pseudogenes remains to be determined.

## Future perspectives

For a long time, pseudogenes were defined as “non-functional copies” sequences of functional gene in the genome. The prevalence and significance of pseudogenes in microorganisms have been increasingly acknowledged and have emerged as a focal point of research in recent years. In this review, we summarize the latest research progress on various aspects of microorganism pseudogenes (Table 3).

**Table 3:** Pseudogenes in microorganisms

	Microorganism	Pseudogene	Parental gene	Parental gene function	Relationship with microorganisms	Reference
Pseudogenes	*S.* Typhi	*ψfepE*	*fepE*	Regulator of O antigen chain length	Pseudogenization of *fepE* contributed to the evade the phagocyte respiratory burst arose by *S.* Typhi	Crawford et al. (2013); Hiyoshi et al. (2018)
	*S.* Paratyphi A	*ψrfbE*	*rfbE*	CDP-tyvelose-2-epimerase, which catalyzes the conversion of O2 to O9	Pseudogenization of *rfbE* contributed to the evade the phagocyte respiratory burst arose by *S.* Paratyphi A	Hiyoshi et al. (2018)
	*S.* Typhi	*ψmarT*	*marT*	Encode by SPI-3, a transcription regulator	Pseudogenization of *marT* contributed to the resistance to H_2_O_2_ of *S.* Typhi	Ortega et al. (2016)
	*S.* Typhi	*ψsseJ*	*sseJ*	SPI-2 T3SS effector, involved in SCV biosynthesis	Pseudogenization of *sseJ* increases the *S. Typhi* cytotoxicity toward epithelial cells	Trombert et al. (2010)
	*S.* Typhi	*ψsopD2*	*sopD2*	SPI-2 T3SS effector, involved in SCV biosynthesis	Pseudogenization of *sopD2* increases the *S. Typhi* cytotoxicity toward epithelial cells	Trombert et al. (2011)
	*S. Typhimurium* ST313	*ψsseI*	*sseI*	SPI-2 T3SS effector, which inhibits DC cell migration	Benefit the systemic infection	Carden et al. (2017)
	*S.* Typhi	*ψsopA*	*sopA*	Effector of SPI-1	Benefit the systemic infection	Ma et al. (2021)
	*S.* Gallinarum *S.* Pullorum	*ψflgK*, *ψflhB*, etc	*flgK*, *flhB*, etc	Involved in the flagella formation	Related to the formation of flagella and phenotypes associated with mobility	Table 2
	*Y. pestis*	*ψyadE*	*yadE*	An autotransporter protein involved in biofilm formation	Pseudogenization of *yadE* contributes to the biofilm stability in *Y. pestis*	Calder et al. (2020)
Functional pseudogenes	*Trypanosoma brucei*	*ψvsg*	*vsg*	Surface glycoprotein	Antigenic variation	Faria et al. (2022)
	*Anaplasma*	*ψmsp2*	*msp2*	Surface antigen	Antigenic variation	Graca et al. (2019); Liu et al. (2019); Rejmanek et al. (2012)
	*Borrelia spp.*	*ψvmp*	*vmp*	Surface antigen	Antigenic variation	Restrepo et al. (1994)
	*Pneumocystis jirovecii*	*ψmsg*	*msg*	Major surface glycoprotein	Antigenic variation	Schmid-Siegert et al. (2017)
	*S.* Typhi	*ψshdA*	*shdA*	Autotransporter adhesin	Product of *ψshdA* function as adhesin	Urrutia et al. (2014)
	*Escherichia coli*	*ψyqiG*	*yqiG*	Has a key role in hydrogen production in *E. coli*	The product of *ψyqiG* is essential for glycolysis in *E. coli*	Zakaria et al. (2018)
	*Brucella melitensis*	*BMEA_B0173*	*BMEA_B0173*	Plays an important role in regulating the function of *Brucella melitensis*	Deletion mutation of the pseudogene BMEA_B0173 in *Brucella melitensis* facilitates colonization in the spleen	Zhang et al. (2022)
	*Trypanosoma brucei*	*-*	*-*	-	Pseudogene-derived small interference RNAs regulate gene expression in African *T. brucei*	Wen et al. (2011)
Others	*M. leprae*	*-*	*-*	-	Intracellular microorganisms have a relatively high proportion of pseudogenes	Cole et al. (2001); Liu et al. (2004)
	*Neisseria meningitidis*	*-*	*-*	-	Intracellular microorganisms have a relatively high proportion of pseudogenes	Liu et al. (2004)
	*Rickettsia*	*-*	*-*	-	Intracellular microorganisms have a relatively high proportion of pseudogenes	Liu et al. (2004)
	*Salmonella* spp.	*-*	*-*	-	Some *Salmonella* serotypes which with a narrow host range have a relatively high proportion of pseudogenes	Table 1
	*Y. pestis*	*-*	*-*	-	The pseudogene profile can be used for *Y. pestis* classification	Tong et al. (2005)

The formation of pseudogenes may be a result of the accumulation of bacterial adaptive evolution (Lawrence et al. 2001; Ochman and Davalos 2006). When the living environment of bacteria changes, such no longer required genes are prone to be lost from the genome by forming pseudogenes. This implies that the type and quantity of pseudogenes can potentially serve as indicators for inferring the evolutionary process of bacteria. Therefore, pseudogenes can be used as evolutionary records, providing very valuable materials for the study of bacterial evolution.

The abundance and distribution of pseudogenes could provide useful information to improve our understanding towards pathogens. For example, the abundance of pseudogenes may be associated with intracellular/free-living lifestyle or host range of the pathogens. And the distribution of pseudogenes (pseudogene profile) can be used for pathogen classification, for example, the classification of *Y. pestis*.

Furthermore, the pseudogenization of some genes contributes to the virulence of certain pathogenic microorganisms, since the complementation of functional parental genes reduces their virulence. It is like a story of “loss is gain,” where loss means the loss of function of the gene through the formation of a pseudogene, and gain means the increase in virulence of the pathogen. The further investigation focusing on pseudogenes holds the potential to enhance our comprehension of bacterial evolution and elucidate the underlying pathogenic mechanisms.

The potential functions of pseudogenes, particularly their contribution to sequence polymorphism, render them a reservoir of sequences for antigenic variation. In addition, greater emphasis should be placed on pseudogenes with potential for transcription or translation, and a more comprehensive exploration of functional pseudogenes is warranted.

Defective genetic mutations (pseudogenes) are also widespread viral genomes, some of which affect the pathogenic characteristic of the virus. More research that focuses on pseudogenes in virus may help to improve our understanding of the evolution and virulence of virus.

It is our hope that more attention and interest can be devoted to the research of pseudogenes in microorganisms. Many efforts and attempts are still required to acquire a comprehensive understanding on pseudogenes and their significance in microorganism, including but not limited to the following: (a) Define the updated and generally accepted concept of pseudogene. (b) Establish novel more accurate pipelines and standards for pseudogene identification. (c) Research on the generation and evolution mechanisms of pseudogenes. (d) Further study the significance of pseudogenization on pathogenicity of various pathogens. (e) Detection of transcribed and translated pseudogenes. (f) Investigation of potential regulatory functions of microbial pseudogenes. (g) Establish database of virus pseudogene and further research. We believe that advanced sequencing technique and bioinformatics tools, in conjunction with genomics, transcriptomics, and proteomics techniques, will improve the accuracy of pseudogene prediction and annotation to fully reveal the secret of pseudogenization in microorganisms.

## Data Availability

Data will be made available on reasonable request.
